# Mapping of Neuro-Cardiac Electrophysiology: Interlinking Epilepsy and Arrhythmia

**DOI:** 10.3390/jcdd10100433

**Published:** 2023-10-18

**Authors:** Sidhartha G. Senapati, Aditi K. Bhanushali, Simmy Lahori, Mridula Sree Naagendran, Shreya Sriram, Arghyadeep Ganguly, Mounika Pusa, Devanshi N. Damani, Kanchan Kulkarni, Shivaram P. Arunachalam

**Affiliations:** 1 Department of Internal Medicine, Texas Tech University Health and Sciences Center, El Paso, TX 79905, USA; senapati.sidhartha36@gmail.com (S.G.S.); damani.devanshi@mayo.edu (D.N.D.); 2 Department of Radiology, Mayo Clinic, Rochester, MN 55905, USA; bhanushali.aditi@mayo.edu (A.K.B.); lahori.simmy@mayo.edu (S.L.); 3 Department of Internal Medicine, University of Connecticut, Farmington, CT 06030, USA; mridulasree1996@gmail.com; 4 Division of Gastroenterology & Hepatology, Mayo Clinic, Rochester, MN 55905, USA; sriram.shreya@mayo.edu; 5 Department of Internal Medicine, Western Michigan University Homer Stryker MD School of Medicine, Kalamazoo, MI 49007, USA; arghyadeepganguly96@gmail.com; 6 Mamata Medical College, Khammam 507002, Telangana, India; mounika.pusa04@gmail.com; 7 Department of Cardiology, Mayo Clinic, Rochester, MN 55905, USA; 8 IHU-LIRYC, Heart Rhythm Disease Institute, Fondation Bordeaux Université, Pessac, 33600 Bordeaux, France; kanchan.kulkarni@ihu-liryc.fr; 9 INSERM, Centre de recherche Cardio-Thoracique de Bordeaux, University of Bordeaux, U1045, 33000 Bordeaux, France; 10 Department of Medicine, Mayo Clinic, Rochester, MN 55905, USA

**Keywords:** epilepsy, arrhythmia, electrophysiology mapping, autonomic nervous system, artificial intelligence

## Abstract

The interplay between neurology and cardiology has gained significant attention in recent years, particularly regarding the shared pathophysiological mechanisms and clinical comorbidities observed in epilepsy and arrhythmias. Neuro-cardiac electrophysiology mapping involves the comprehensive assessment of both neural and cardiac electrical activity, aiming to unravel the intricate connections and potential cross-talk between the brain and the heart. The emergence of artificial intelligence (AI) has revolutionized the field by enabling the analysis of large-scale data sets, complex signal processing, and predictive modeling. AI algorithms have been applied to neuroimaging, electroencephalography (EEG), electrocardiography (ECG), and other diagnostic modalities to identify subtle patterns, classify disease subtypes, predict outcomes, and guide personalized treatment strategies. In this review, we highlight the potential clinical implications of neuro-cardiac mapping and AI in the management of epilepsy and arrhythmias. We address the challenges and limitations associated with these approaches, including data quality, interpretability, and ethical considerations. Further research and collaboration between neurologists, cardiologists, and AI experts are needed to fully unlock the potential of this interdisciplinary field.

## 1. Introduction

The intricate relationship between the nervous and cardiovascular systems has long fascinated researchers and clinicians. Recent advancements in neuro-cardiac electrophysiology have shed light on the interconnected nature of epilepsy and arrhythmias, two complex disorders affecting the brain and heart, respectively. The mapping of neuro-cardiac electrophysiology has emerged as a promising avenue for unraveling the shared mechanisms, common etiological factors, and potential therapeutic interventions that bridge these seemingly disparate conditions.

Epilepsy, characterized by recurrent seizures originating from abnormal electrical activity in the brain, affects approximately 70 million people worldwide [1]. Arrhythmias, on the other hand, encompass a spectrum of irregular heart rhythms, ranging from benign palpitations to life-threatening conditions such as ventricular fibrillation (VF) [2]. While epilepsy and arrhythmias have traditionally been studied and managed as distinct entities, emerging evidence suggests a bidirectional relationship between the two, with shared pathophysiological pathways and overlapping risk factors.

Epilepsy is a neurological disease characterized by spontaneous repetitive seizures that result in an excessive abnormal electrical discharge in the cortical neurons [3]. This phenomenon causes a sympathetic nervous system (SNS) predominance and a subsequent catecholamine surge, which is known to trigger abnormal cardiac electrical activity known as arrhythmias [4]. Some studies suggest the concept of the lateralization of the insular cortex with respect to cardiac function. The right insula is more involved in sympathetic cardiac regulation whereas the left insula is more involved in parasympathetic regulation [5]. Seizures, which are abnormal electrical discharges in the brain, can, indeed, impact the autonomic nervous system and potentially lead to cardiac arrhythmias. The specific mechanisms are complex and can involve various brain regions, including the insula. Seizures can trigger changes in sympathetic and parasympathetic activity, leading to alterations in heart rate and rhythm. This suggests that the mechanism of cardiac arrhythmias may depend on the region involved during a seizure. Arrhythmias can be divided into two categories: bradyarrhythmias (slow heart rhythms) and tachyarrhythmias (fast heart rhythms). Bradyarrhythmias can include sinus bradycardia, atrioventricular block, and asystole. Tachyarrhythmias can include ventricular tachycardia (VT), VF, and atrial fibrillation (AF).

According to a nationwide inpatient analysis by Desai et al., the most frequent arrhythmia in epilepsy patients was AF [6]. Distinct arrhythmias may manifest during the ictal, postictal, and interictal phases of epilepsy. Ictal asystole is the most commonly identified cardiac arrhythmia during the ictal phase, whereas sinus tachycardia, bradycardia, atrioventricular (AV) block, and AF have also been reported [4,7]. In the postictal phase, patients can develop asystole, bradycardia, AV block, AF, atrial flutter, VT, or VF [4,8]. Postictal arrhythmias were mostly found to be associated with SUDEP (Sudden Unexplained Death in Epilepsy) [4]. In addition to this, epilepsy has been shown to have a correlation with long QT syndrome (LQTS) due to ion channel mutations. Overall, epileptic patients have a higher prevalence of arrhythmias than the general population [8]. 

In this review, we describe the correlation between epilepsy and cardiac arrhythmias. We provide an overview of the various arrhythmias, their prevalence, and associated seizure types, characterize the pathophysiological mechanism for the onset of arrhythmias in epilepsy patients, and present insights into which types of arrhythmias predispose an individual to higher rates of SUDEP. We also provide a comprehensive summary of contemporary neuro-cardiac electrophysiology studies, focusing specifically on the interplay between epilepsy and arrhythmias, and seeking to establish a unified framework for understanding the complex relationship between the brain and the heart. Furthermore, we address the potential of AI-driven advancements in clinical practice and highlight avenues for further research. Harnessing the power of AI in uncovering the shared pathophysiology and mechanisms underlying epilepsy and arrhythmia can enable personalized treatment strategies and targeted interventions that optimize patient outcomes.

## 2. Brain–Heart Interplay

The autonomic nervous system (ANS) plays a vital role in regulating cardiac electrophysiological functions and in triggering conditions that cause cardiac dysregulation. It comprises the sympathetic (SNS) and parasympathetic nervous systems (PNS), which interact using a complex network of intrinsic cardiac nerves and ganglia, stellate ganglia, the spinal cord, the brainstem, and the higher brain centers, thus regulating the ANS [9,10,11,12]. The order of the neuro-cardiac interconnections has been described as a three-level hierarchy. It starts from the central nervous system (CNS), specifically the insular cortex, extending to the medulla and to the intrinsic cardiac nervous system (ICNS) [13,14]. This Level 1 of the hierarchy includes the higher cortical centers, the brainstem, and the spinal cord. The intrathoracic extracardiac neurons and the stellate ganglia form Level 2, while Level 3 is the ICNS [13,14].

The ICNS pathways comprise a network of neurons and ganglionated-plexi (GPs) present within the epicardial fat pads. They act as a relay station that processes information from the heart and trans-conducts the local signals to regulate cardiac function, independent of the higher ANS [15]. The ICNS is sometimes referred to as the heart’s “little brain” [14]. The ICNS consists of efferent parasympathetic and sympathetic neurons, afferent sensory neurons, local neurons, and interneurons acting via the diverse neurotransmitters [16]. The epicardial ganglia are distributed widely and their sizes range from those that are microscopic to those that are discernible with the naked eye. In addition, afferent neurons may mediate the reverse heart-to-brain communication by continuously sending information to the CNS, which impacts the neuronal circuits involved in perception, cognition, and emotional processing [17].

The extrinsic cardiac nervous system mediates information between the heart and the cervical, stellate, and thoracic ganglia forming sympathetic connections, and the medulla oblongata forming the parasympathetic connections [15]. The ganglia of the parasympathetic division are distributed mainly within the epicardial area and are intrinsic, while the ganglia of the sympathetic division are present in the sympathetic chain or in the paravertebral ganglia [18,19,20].

The heart receives sympathetic innervation through a complex network of nerve fibers. The sympathetic nerves responsible for innervating the heart originate from paravertebral ganglia, which are located adjacent to the spinal cord. Of particular importance in the sympathetic innervation of the heart are the superior thoracic ganglia, also known as stellate ganglia, which are situated in the lower neck region near the base of the cervical spine. The postganglionic fibers arising from the stellate ganglia form a complex network called the cardiac plexus. This intricate network extends along the anterior surface of the tracheal bifurcation, behind the ascending aorta. From the cardiac plexus, sympathetic nerve fibers project to different regions of the heart, including the myocardium (heart muscle) and specialized structures such as the sinoatrial (SA) node and AV node.

Upon activation, sympathetic nerves release norepinephrine (noradrenaline) as the main neurotransmitter. Norepinephrine binds to β-1 adrenergic receptors located in the heart. This binding triggers a cascade of intracellular events, leading to increased calcium influx into cardiac muscle cells during each action potential. The elevated intracellular calcium levels enhance the contractility of the myocardium, resulting in a more forceful and efficient contraction of the heart (positive inotropic effect) [21].

The parasympathetic effect on the heart is mediated by the M2 receptors [22]. The PNS comprises the parasympathetic preganglionic neurons located in the medulla oblongata within and ventrolateral to the nucleus ambiguous (NAmb) as well as in the dorsal motor nucleus of the vagus (DMNX) and the reticular formation between the two nuclei. Parasympathetic postganglionic fibers innervate the cardiac conduction system along with the atrial and ventricular working myocardium, which releases Acetylcholine and vasoactive intestinal peptide [23,24]. When the Acetylcholine released by the vagus nerves binds to the M2 muscarinic receptors it significantly slows heart rates (mediated by G-protein inwardly rectifying potassium [GIRK] channels), shortens atrial action potentials, increases smooth muscle contraction, inhibits the funny current, activates the IKACh channel, and decreases contractility [22], which produces negative chronotropic, negative homotopy as well as negative inotropy and lusitropy in the heart [23,24].

**Figure 1:**
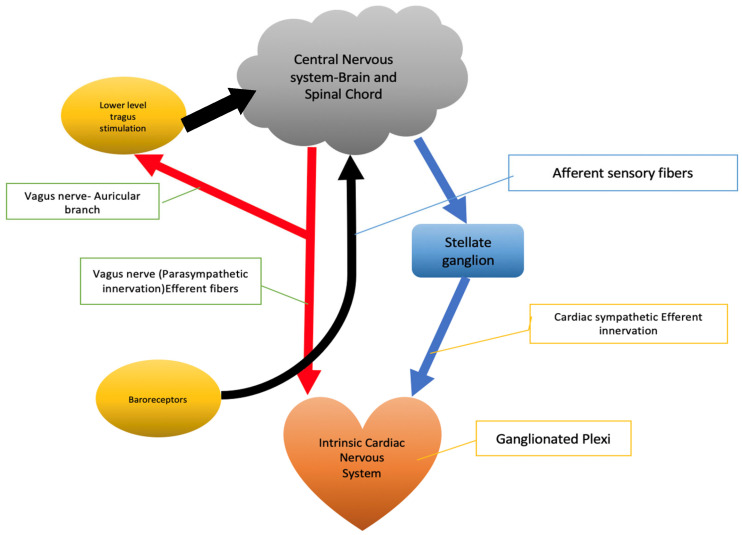
Brain–heart interplay.

Under normal physiological conditions, the heart is controlled by a fine balance of parasympathetic and sympathetic signals, the peripheral nervous system, and the CNS [25]. The role of parasympathetic innervation is more significant in the atria compared to the ventricles. Additionally, the sympathetic innervation of the ventricles is more influential than the parasympathetic innervation of the ventricles. However, autonomic imbalance has been associated with various cardiovascular abnormalities (Figure 1).

## 3. Ictal Arrhythmias

Cardiac rhythm changes often accompany seizures, and the most common among them are ictal tachycardia (IT) and ictal bradycardia (IB) [26], although other forms of rhythm alterations have been described in the literature. The increasing use of electroencephalogram (EEG), electrocardiogram (ECG), and video EEG (vEEG) monitoring has recently resulted in a greater recognition and characterization of interictal cardiac arrhythmias [27].

### 3.1. Pathophysiology of Heart Rate Changes in Epilepsy

The ANS control of the cardiovascular system is multifocal. Alterations in heart rate (HR) take place in distinct regions of the brain, such as the prefrontal cortex, insular cortex, brainstem, thalamus, hypothalamus, and the limbic system, which includes the cingulate gyrus and amygdala [28,29]. If there is an epileptic focus in any of the structures, it could lead to an arrhythmia [30]. Oppenheimer et al. have shown that the left-sided stimulation of the insular cortex leads to an HR increase, whereas the stimulation of the right-sided insular cortex causes a deceleration in the HR [31]. Thornton and associates have reported that the stimulation of the midbrain could lead to increased HR [32]. In 2007, Leung et al. demonstrated a decrease in HR upon stimulation of the left cingulate gyrus, which may lead to asystole [33].

The major mechanisms through which seizures can trigger an arrhythmia are altered cardiac electrophysiological parameters, ion channel abnormalities, and autonomic imbalance [34]. IT (HR > 100 bpm) is caused due to the increased sympathetic stimulation of the heart. Tachycardia leads to increased myocardial oxygen demand and a higher risk of cardiovascular mortality [26,35]. IB (HR < 50 bpm) is caused by a sympatho-vagal imbalance wherein the PNS is in overdrive. Ictal asystole (IA) is a subtype of IB wherein there is an absence of QRS complexes for longer than 4 s with seizure onset. IA often leads to a cardiac “vagal storm” and may cause a loss of consciousness and subsequent injuries [4]. Chronic epilepsy often leads to secondary cardiac ion channelopathies due to long-term alterations in the ANS control of the heart [36]. Channelopathies in the cardiac conduction system can lead to alterations in the transmission of action potentials and adversely affect the pacemaker activity and AV nodal conduction, leading to arrhythmias. A commonly associated channelopathy in epilepsy and SUDEP is LQTS, which can affect voltage-gated K^+^ channels, ryanodine receptors, and voltage-gated Na^+^ channels [37,38]. The altered ion channels in the cardiac action potential can lead to abnormal Na^+^ and K^+^ currents during the plateau phase, resulting in several changes in the electrical activity of the heart. These changes include an increase in cytosolic Ca^2+^ content, the prolongation of the ventricular action potential, and delayed repolarization. These alterations in the electrical properties of the heart can predispose individuals to ventricular arrhythmias and increase the risk of sudden cardiac death [39]. Finally, partial and generalized seizures may induce chronic alterations in the central autonomic network (which includes the insular cortex, amygdala, and periaqueductal grey matter). The altered autonomic firing in the central autonomic tone due to long-term epilepsy may induce downstream changes in the medullary autonomic structures, mainly the nucleus tractus solitarius and nucleus ambiguous [40], which, in turn, may lead to abnormal cardiac action potential generation and arrhythmias.

### 3.2. Ictal Tachycardia

IT is commonly defined as a HR in excess of 100 bpm [28]. However, some studies have included differing definitions, including HR > 120 bpm and HR > 10 bpm above the baseline HR [35]. Several studies over the years have attempted to characterize its prevalence in both small and large groups of epilepsy patients [41,42,43]. Egglestone et al. reported that the cumulative weighted average percentage of patients with IT was 82% [35]. IT may begin during a seizure [44] but may also commence in the moments preceding the episode of seizure [45,46]. While IT is predominantly composed of sinus tachycardia [28], in some cases, AF/flutter and VT may occur. Van der Linde and associates reported 13 cases of ictal AF, 1 case of ictal atrial flutter, and 3 cases of ictal VF in 17 cases of observed tachycardia associated with seizures. However, it is important to be aware that only 5 of the 17 subjects reported to demonstrate IT had an ictal [GA2] arrhythmia as detected by video EEG (which is more sensitive and accurate than conventional EEG) [4].

With regards to seizures that accompany IT and its variants, both partial and generalized seizures have been associated with IT episodes. Partial seizures, both with and without secondary generalization, resulted in tachycardia in a cumulative average of 71% across the studies reviewed by Egglestone and colleagues [35], with the lowest percentage in the report by Garcia et al. being 32.9% [44] to 100% [97 events in 38 pts (18 M/20 F); aged 3–53 yrs (mean: 27)] being reported in others [47,48]. The same review examined seven studies and found that generalized seizures were associated with 64% of IT episodes. While the above review found that partial seizures have a greater association with IT/HR changes, some studies have documented that generalized seizures are associated with a higher risk of IT compared to partial seizures [49,50]. These studies also found that there is a predisposition to developing IT in cases of partial seizures that ultimately result in a secondary generalization. Opherk et al. studied HR and ECG changes in 102 cases of seizures (71 non-generalized and 31 generalized) from 41 patients and found that ictal HR was significantly higher in generalized seizures vs. non generalized seizures (*p* < 0.03) [50].

With regards to the locations of epileptic foci that predispose to IT, seizures arising from the right side of the insular cortex are more strongly associated with the development of IT [31,45]. Furthermore, studies have also reported increased HR changes with seizures of temporal origin vs. those of extra-temporal origin [44,51]. Garcia et al. analyzed 100 seizures from 38 patients, whereas Weil et al. analyzed vEEG data from 21 patients with epilepsy over 24 h [51]. In these studies, compared to 22% and 11% of extratemporal seizures, it was observed that 78% and 62% of seizures with temporal lobe onset were related to an increase in HR, respectively [44,51]. In another study, it was demonstrated that, out of 90 partial seizures, 56 had a temporal lobe origin, 29 had a frontal origin, and 5 had an unknown origin [52]. Of the 90 seizures, 44 (49%) had an association with early HR increase (early HR being defined as the HR in the first 10 s of recorded ictal discharge on EEG) [52]. Notably, the authors reported that 50% (22/44) of the seizures in which early HR was increased were of temporal lobe origin, while 80% (18/23) of the seizures where there was a decrease in early HR were of temporal lobe origin. Their data suggest that temporal-lobe-based partial seizures play an important role in HR changes, and may be associated with both increase and decrease in HR [52].

### 3.3. Ictal Bradycardia

Conventionally defined as HR < 50 bpm, IB is thought to be much rarer compared to IT and affects less than 5% of epilepsy patients [4,28,53]. However, the recognition of IB and its subtypes is important because it could potentially lead to life-threatening complications and SUDEP [53]. The types of IB can be classified as IA, ictal sinus bradycardia, and ictal AV-conduction block [4]. In contrast to IT, IB usually starts 10–30 s following the onset of seizures on EEG [27,54], although some authors have reported IBs preceding [55] and starting simultaneously [56] with the seizure episode. Nonetheless, the typical course of an episode of IB starts with initial sinus tachycardia followed by a progressive decrease in the HR, occasionally terminating in an episode of asystole of variable length [53]. This is important, as most of these asystolic episodes last for 3–20 s and are not life-threatening, but, sometimes, longer pauses may result in SUDEP. Van der Lende et al. studied a total of 164 cases of IB in their systematic review and documented 103 cases of IA (with a further 13 being postictal asystoles) [4]. They also compiled 25 cases of IB without an asystolic episode via vEEG, [GA2] and a further 11 cases of ictal AV block (with 9 being complete AV blocks and 2 being second-degree AV blocks) [4].

A consistent finding in all subtypes of IB is that they almost always occur in people suffering from focal dyscognitive seizures (FDS), which is also called a complex partial seizure. It was also strongly associated with an origin in the temporal lobe [4,28,53]. A comprehensive study of 103 cases of IA demonstrated that 99% were associated with FDS, 25 cases of IB without asystole had 100% association with FDS, and, of the 11 cases of ictal AV block, 90% were associated with FDS [57]. Clinically, however, there is a high chance of IBs being missed, because the association with loss of cognition, awareness, and muscle tone makes these episodes hard to distinguish from syncope.

## 4. Postictal Arrhythmia

Postictal arrhythmias are abnormal cardiac rhythms that occur after a seizure due to autonomic dysregulation, typically characterized by increased sympathetic and decreased parasympathetic output in the early postictal phase and impaired vagal recovery seen in the late phase. These changes may generate fatal arrhythmias and lead to SUDEP [4,58,59]. Moreover, the direct activation of central autonomic networks may be the cause of the link between arrhythmia and seizures [57,60,61]. When compared to the general population, people with epilepsy were found to have a three-fold higher risk of developing VT/VF [62]. Most cases of VT/VF in epilepsy were, however, not seizure-related and were probably related to cardiovascular comorbidities. Nevertheless, in a subset of cases, seizure-induced VF may have played a role. However, the exact mechanism underlying the increased risk of ventricular tachycardia (VT) or ventricular fibrillation (VF) in people with epilepsy is not fully understood. Some potential factors that could contribute to this association include a rise in catecholamines, and various other factors may contribute to postictal VF, including a higher prevalence of ECG markers for sudden cardiac arrest, peri-ictal QTc prolongation, ST changes, and increased troponin levels [62].

Convulsive seizures were associated with the detection of postictal asystole, AF, and VF [4]. Postictal VF is always categorized as (near) SUDEP, and postictal asystole is commonly linked to SUDEP [63]. Postictal generalized electrographic suppression (PGES) and apnea were the main triggers of the majority of postictal asystoles [4]. Prolonged apnea activates the carotid chemoreceptors, resulting in arousal, and, ultimately, vagally mediated bradycardia or even cardiac collapse [58,64]. These statements highlight the complex relationship between convulsive seizures, postictal events, and cardiac complications, particularly in the context of SUDEP. It is important to note that SUDEP is rare but has significant implications for individuals with epilepsy.

Postictal AF, although rarely reported, is a highly dangerous complication of Generalized Tonic-Clonic Seizure (GTCS). It is associated with PGES and autonomic dysregulation [58]. About 21 documented cases of postictal AF were found in the literature, with 19 cases being associated with GTCS and 2 with focal seizures [65]. Prolonged PGES, medically refractory epilepsy, and genetic abnormalities like sodium (SCN1A) and potassium (KCNA1) channelopathies highly increase the risk of developing SUDEP [65,66]. The decreased ventricular output seen in AF could be a precipitating factor for SUDEP [58].

## 5. Long QT Syndrome and Epilepsy

LQTS is a hereditary condition that affects the electrical activity of the heart and raises the risk of irregular heartbeats. It affects about 1 in every 2500 individuals, and symptoms typically appear in children and adolescents. If left untreated, the 10-year mortality rate among symptomatic individuals can be as high as 50% [67]. The prevalence of LQT2 is higher at 3.7% compared to other LQTS variants (0.7%) in the general population.

There is said to be a genetic interplay between LQTS and an increased risk of epilepsy. These genes include the following: KCNQ1, associated with LQTS type 1; KCNH2, associated with LQTS type 2; SCN5A gene, associated with LQTS type 3; and ANK2, associated with LQTS type 4 [68].

The pathophysiology between the two is explained by a few theories. One theory for the cause of epilepsy in LQTS is that mutations in the ion channel genes that control calcium and potassium currents cause aberrant calcium fluxes and neuronal hyper-excitability. This may cause abnormal electrical activity to be generated, presenting as seizures. The second theory is suggestive of dysfunction of the ANS in LQTS due to ion channel mutations that can lead to the abnormal regulation of cardiac and neuronal activity, potentially contributing to seizures. The third theory suggests that prolonged reduction in blood flow to the brain leads to epilepsy because of an irregular heart rhythm [69].

To diagnose LQTS, it is important to identify key symptoms in the patient’s medical history, such as sudden syncope, near syncope, or prolonged syncope with seizures [70], and to carry out a standard 12-lead ECG test. The ECG test should be repeated if the frequency or nature of the seizures changes or if the seizures do not respond to the therapy [70]. If a sudden loss of consciousness is followed by myoclonic jerks, arrhythmias such as bradycardia or torsade de pointes ventricular tachycardia should be considered. The key indicators that may help differentiate between LQTS and epilepsy in pediatric age groups include brief seizure episodes with no postictal drowsiness, syncope, and a strong family history of LQTS [69].

Misdiagnosis can be avoided by some ECG and EEG indicators. Prolonged QT interval, torsades de pointes, and bradyarrhythmias in ECG with epileptiform discharges, interictal abnormalities, and seizure activity in an EEG are usually suggestive of epilepsy [71]. Both the test findings along with a comprehensive clinical evaluation are necessary for an accurate diagnosis. Machine learning algorithms and AI tools can be utilized to help better analyze the data.

As many anti-epileptic drugs (AEDs) have the potential to worsen QT prolongation and increase the risk of cardiac arrhythmias, the AEDs that have been associated with QT prolongation and increased risk of cardiac arrhythmias include the following: (1) some Sodium Channel Blockers—AEDs that block sodium channels, like Flecainide and Procainamide, can slow down cardiac repolarization and prolong the QT interval; (2) some Potassium Channel Blockers—certain AEDs, such as Sotalol and Amiodarone, block potassium channels, which can delay potassium efflux during repolarization; (3) some Calcium Channel Blockers—AEDs like Verapamil and Diltiazem, which block calcium channels, can affect the timing of calcium ion flow during the action potential; and (4) Multiple Drug Interactions—in some cases, combining multiple AEDs or combining AEDs with other medications may increase the risk of QT prolongation. Choosing an appropriate mode of therapy is necessary. The treatment options for epilepsy in LQTS patients include the following: (1) non-pharmacological therapies such as vagus nerve stimulation, ketogenic diet, or surgery; (2) the use of specific AEDs which are less likely to cause QT prolongation, like lamotrigine, levetiracetam, and topiramate, and the avoidance of high-risk AEDs such as phenytoin, carbamazepine, and valproate; and (3) close monitoring for cardiac events for patients on medication [72]. A multidisciplinary approach is essential to ensure the best possible outcomes for patients.

## 6. Sudden Unexplained Death in Epilepsy

Compared to the general population, people with epilepsy have a higher risk of death, with SUDEP being the main direct epilepsy-related cause of premature death [60]. SUDEP is described as the sudden and unexpected death of a person with epilepsy that is not related to trauma or drowning and occurs without a toxicological or anatomical cause of death being found during the post-mortem examination. Seizures are typically the cause of SUDEP, and changes in cardiorespiratory function brought on by seizures are the most likely precipitating factor [73]. The incidence of SUDEP in the general epilepsy population ranges from 1.2 to 1.3 per 1000 person-year, with a peak incidence among teenagers and young adults (under 45 years) [74].

Although the pathology is still poorly understood, research has established that most cases of SUDEP are a result of postictal dysfunction in the central respiratory center that ultimately leads to terminal apnea and cardiac arrest. However, interictal SUDEP is most likely associated with episodes of IA and VF [29,43,75]. Some studies, however, have found that IA may have a protective effect against SUDEP [76]; however, further studies are required to validate these findings.

## 7. Discussion

Epilepsy, a neurological condition, is defined by meeting any of the following criteria: experiencing at least two unprovoked seizures that are separated by more than 24 h, having experienced a single unprovoked seizure along with a 60% chance of having a recurrent seizure within the next decade, or having received a clinical diagnosis of epilepsy [77,78,79]. SUDEP refers to the sudden and unexpected death of individuals who had a prior diagnosis of epilepsy, and were in a healthy condition, but for which an autopsy could not determine the cause of death. The factors increasing the likelihood of SUDEP are (a) the sleep state, (b) seizures (most commonly General Tonic-Clonic Seizures), and (c) seizure clusters. The increased vagal tone and autonomic instability during the above-mentioned conditions are the factors responsible for SUDEP. The likelihood of sudden death resulting from arrhythmia is higher in individuals who already have pre-existing structural heart diseases [61].

Syncope and psychogenic events are the two most common forms of non-epileptic paroxysmal events (NEPEs). Misdiagnosed epileptic cases account for 20–30% of all the diagnoses [79,80]. Hence, the accurate diagnosis of NEPE becomes important before the initiation of long and rigorous epileptic treatment. Simultaneous EEG and ECG become important in the early diagnosis of patients with epilepsy or convulsive syncope and further possible arrhythmic episodes. Epilepsy is also known to cause cardiac syncope through secondary tachycardia and bradycardia mediated via the ANS [81].

The concurrent recording of an Electrocardiogram (ECG) and an Electroencephalogram (EEG) is a crucial clinical requirement to identify and understand the coexisting abnormalities in both heart and brain functions. This combined testing helps determine the clinical significance of ECG and EEG abnormalities. It is particularly useful in distinguishing potentially rare life-threatening causes of fainting (syncope) that might mimic seizures or conditions that could lead to resistance to antiepileptic therapy [82]. Some cardiological conditions which may coexist with seizures, and that warrant simultaneous ECG and EEG recordings, are as follows:

A LQTS: Simultaneous EEG and ECG recording becomes important in detecting the QT interval and T wave abnormalities, and in the evaluation of convulsive syncope [83].
B Brugada syndrome: An arrhythmic disorder that may coexist with epileptic disorder. Simultaneous ECG and EEG recording can help in distinguishing arrhythmic from epileptic forms, as epileptic forms carry a higher risk of sudden death [84].
C Myocardial-ischemia-related ST segment changes: Ventricular arrhythmia may precede the development of ST segment changes during myocardial ischemia. It is sometimes associated with syncope and can be monitored using simultaneous EEG and ECG recording.

EEG detects the neural activity generated by the cortical pyramidal neurons that are located perpendicular to the surface of the brain. It is the summation of the excitatory and inhibitory postsynaptic signals synchronously fired by the neurons [85]. Surface EEG and accurate annotation is the gold standard method for diagnosing epilepsy [86]. Delayed diagnosis or misdiagnosis can result in serious consequences [86,87]. False positives can result in the unnecessary prescription of medications. Recording simultaneous ECG and EEG is crucial as it not only helps identify the cardiac rhythm abnormalities that may be present during an epileptic episode but also allows for the detection of the clinical features and rhythm abnormalities associated with seizures. The early prediction of seizures has also been made possible by studying the EEG and ECG simultaneously [88,89,90,91,92], for example, in predicting events of temporal lobe epilepsy by analyzing heart rate variability (HRV) in children. An early decrease in HR was found to be associated with temporal lobe seizures [42]. ECG recordings can also provide markers of seizure events that may not be detectable by EEG [52,93]. The side effects caused by antiepileptic drugs like lethal cardiac arrhythmia, or AV conduction block could be observed through simultaneous EEG and ECG monitoring. This enables the neurologist to detect abnormal resting ECG and serious cardiac rhythm disturbances [81].

While simultaneous ECG recording with EEG is necessary in clinical settings to detect and clarify abnormalities in both EEG and ECG, analyzing long recordings can be a time-consuming process. The possible reasons for the abnormally high number of false positives include a lack of formal standards or mandatory EEG training. The difficulties in reading and interpreting EEG might be missed upon clinician-reading, as a delayed diagnosis can also delay the initiation of treatment in patients and impact outcomes. This issue is also compounded by a treatment gap attributed to inequalities in distribution and access to healthcare. Practicing it every time a patient presents with a history of seizure or arrhythmia can be cumbersome and can lead to the unnecessary utilization of resources.

**Figure 2:**
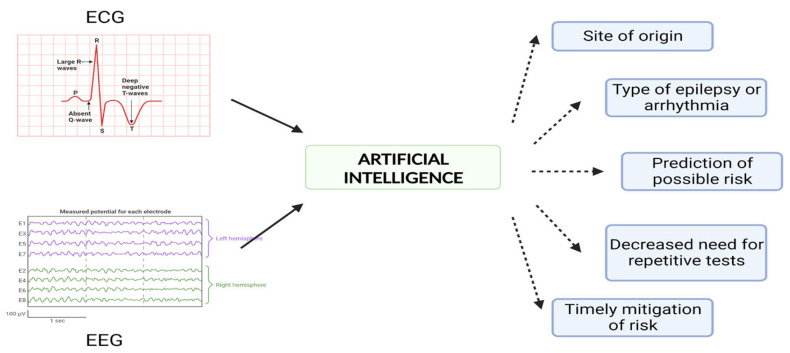
Potential of artificial intelligence in epilepsy and arrhythmia diagnoses.

The use of AI-based analysis in ECGs has demonstrated encouraging outcomes in the diagnosis of hypertrophic cardiomyopathy, in the screening of first-degree relatives of patients with dilated cardiomyopathy, and in the detection of left ventricular dysfunction [94,95,96]. By analyzing ECG signals, AI algorithms can help identify the specific site of origin of certain arrhythmias in the myocardium [97]. Similar success has been recorded in the field of neurology in analyzing EEG results, localizing epileptic regions, and predicting the surgical outcomes of epilepsy [97]. Hence, AI can minimize the need for repeated EEG and ECG measurements and assist in the diagnosis and prediction of impending seizures and arrhythmias. Factors like patient demographics, type of seizure or arrhythmia, data collection, and storage methods can all enhance detection using AI. Categorizing patients based on the likelihood of experiencing seizures would give us an edge in putting patients on prophylactic antiepileptic medications. Analyzing the different kinds of seizures would give us warning about the type of impending arrhythmia and enable the timely mitigation of risk (Figure 2).

However, despite its promising potential, some challenges are being anticipated while clinically implementing AI. The involvement of AI in the decision-making process can challenge patients’ trust in their physicians [98]. Professional liability, in cases of incorrect decisions being made, is still under question. Moreover, physicians who lack experience in AI may fail to understand its algorithms. Furthermore, medical malpractice may become more complicated as different stakeholders will be involved. Incorporating the technology could potentially reduce the time and cost associated with diagnostic tests and monitoring devices. However, there is currently insufficient evidence to support the cost-effectiveness of the system.

As a result of the significant dependence on electronic health record systems, it is possible that some data may be missing. People from certain economic classes may not have access to healthcare services or diagnostic tests. In such scenarios, AI may interpret the lack of data as a low disease burden in this group of the population. Another potential challenge is the interpretation of the EEG and ECG records, as they might be stored in different formats, or can be ambiguous, heterogeneous, or incomplete. This can lead to data sparsity and redundancy. Cybersecurity is another challenge that needs to be addressed. As they are potential targets for hacking, regulatory frameworks are put in place. Machine learning apps, like Apple health apps, have to comply with these rules as they put individuals’ privacy at risk [99].

## 8. Conclusions

Mapping neuro-cardiac electrophysiology has yielded valuable insights into the connection between epilepsy and arrhythmia. Advanced imaging techniques, like EEG and ECG, have identified shared pathways involving abnormal neural activity, autonomic dysfunction, and structural abnormalities. This understanding has significant implications for diagnosing and managing patients with epilepsy and arrhythmia, enabling comprehensive evaluations and guiding treatment decisions. Collaboration among healthcare professionals is crucial in delivering holistic care for these conditions. Despite this progress, further research is needed to uncover the precise mechanisms and develop personalized therapies. The AI analysis of EEGs and ECGs shows promise in improving diagnosis and management, benefiting both patients and healthcare providers. Future work should address the limitations and assess cost-effectiveness. Ultimately, AI-assisted decision-making holds the potential to revolutionize diagnostic and prognostic services in healthcare.

## Data Availability

The review was based on publicly available academic literature databases.
